# Genetic silencing of sGC β1 in cancer: role of epigenetic regulation

**DOI:** 10.1186/2050-6511-14-S1-P66

**Published:** 2013-08-29

**Authors:** Alex Sotolongo, Ka Bian, Ferid Murad

**Affiliations:** 1 Department of Biochemistry and molecular Medicine, George Washington University, 2300 I street, NW; Ross Hall 543, Washington, DC 20037, USA

## Background

The nitric oxide (NO) receptor soluble guanylyl cyclase (sGC) is composed of two subunits of α1,2 and β1,2, sGCα1β1 being the major heterodimer that catalyzes the formation of second messenger cGMP. NO-cGMP signaling plays a critical role in numerous processes, and retarded signaling function by either limited NO bioavailability, or decrease in expression or activity of sGC correlates with several disease states including hypertension, neurodegenerative disorders, inflammation, and cancer. Our analysis of human databases revealed a statistically significant reduction of sGC transcript levels in multiple human cancer specimens including glioma and breast cancers. We have recently demonstrated that restoration of sGC expression levels in glioma cell lines significantly reduced cell proliferation, colony formation, and growth of orthotopically implanted glioma cells in mice, suggesting a potent anti-tumor property for the sGC. However, the mechanism underlying the genetic silencing of sGC in cancer is unknown.

## Results and discussion

Here we report that sGCβ1 is regulated epigenetically by histone acetylation in breast and lung cancers cell lines. Treatment with HDAC inhibitors LBH-589 (panobinostat), MS-275 (entinostat) and Trichostatin-A were able to increase expression levels of sGCβ1 up to 25 fold above control in the triple negative breast cancer cell line MDA-MB-231. However, the treatment of cells with histone lysine demethylase inhibitor (BIX-0192); the histone lysine demethylase inhibitor (trans-2-PCPA), and DNA methylation inhibitors (5-aza-2’-deoxycytidine and chlorogenic acid) had no effect on sGCβ1 expression. Thus, histone acetylation plays an influential role in regulating sGCβ1 expression, while other epigenetic marks such as DNA methylation, and histone methylation do not. Over expression of histone deacetylase (HDAC) isoforms 1 and 3 significantly reduced sGCβ1 expression, and pharmacological inhibition of histone acetyl transferase (HAT) p300/pCAF achieved a similar result. Further analysis of the molecular aspects of sGCβ1 epigenetic regulation should offer opportunities to target diseases, such as cancer, that are marked by decreased sGCβ1 expression.

